# GAC-Net: A Geometric–Attention Fusion Network for Sparse Depth Completion from LiDAR and Image

**DOI:** 10.3390/s25175495

**Published:** 2025-09-04

**Authors:** Xingli Gan, Kuang Zhu, Min Sun, Leyang Zhao, Canwei Lai

**Affiliations:** 1 School of Computer Science and Technology, Zhejiang University of Science and Technology, Hangzhou 310023, China; 212308802004@zust.edu.cn (K.Z.); 222308855053@zust.edu.cn (M.S.); 222408855059@zust.edu.cn (C.L.); 2 School of Mathematics and Statistics, University of New South Wales, Sydney, NSW 2052, Australia; z5167073@ad.unsw.edu.au

**Keywords:** depth completion, sparse LiDAR, deep learning, computer vision, 3D geometric representation, point cloud representation, attention-based fusion

## Abstract

Depth completion aims to reconstruct dense depth maps from sparse LiDAR measurements guided by RGB images. Although BPNet enhanced depth structure perception through a bilateral propagation module and achieved state-of-the-art performance at the time, there is still room for improvement in leveraging 3D geometric priors and adaptively fusing heterogeneous modalities. To this end, we proposed GAC-Net, a Geometric–Attention Fusion Network that enhances geometric representation and cross-modal fusion. Specifically, we designed a dual-branch PointNet++-S encoder, where two PointNet++ modules with different receptive fields are applied to extract scale-aware geometric features from the back-projected sparse point cloud. These features are then fused using a channel attention mechanism to form a robust global 3D representation. A Channel Attention-Based Feature Fusion Module (CAFFM) was further introduced to adaptively integrate this geometric prior with RGB and depth features. Experiments on the KITTI depth completion benchmark demonstrated the effectiveness of GAC-Net, achieving an RMSE of 680.82 mm, ranking first among all peer-reviewed methods at the time of submission.

## 1. Introduction

Depth completion [1] is a fundamental perception task that aims to reconstruct dense and accurate depth maps from sparse sensor inputs. It plays a critical role in various applications, such as autonomous driving [2,3,4,5,6,7,8,9,10,11] and sensor-based environmental understanding [12,13]. However, due to hardware constraints and occlusions, LiDAR sensors typically produce sparse and unevenly distributed depth maps. For example, a 64-line LiDAR provides valid measurements for only about 4% of image pixels [14,15,16], which poses significant challenges for downstream 3D perception tasks [17]. Although the KITTI dataset [18] remains the most widely used benchmark for depth completion, recent works such as RSUD20K [19] emphasize the importance of dataset diversity and robustness evaluation under diverse conditions, further underscoring the need for methods with strong generalization ability.

To overcome this challenge, mainstream approaches [20,21] typically adopt multi-modal fusion strategies by combining sparse depth maps with RGB images. Many existing methods [22,23,24] follow a two-stage architecture that performs early or late fusion of depth and image features. However, these approaches struggle with distinguishing between valid and missing observations and often fail to effectively process the irregular distribution of sparse depth values [25]. A representative improvement, BPNet [26], introduces a three-stage framework that begins with a bilateral propagation module to generate an initial dense depth prior, followed by a multi-modal fusion stage and a final refinement stage. Nevertheless, its fusion module relies on a conventional U-Net backbone [27] and lacks explicit modeling of 3D geometric priors and adaptive integration of heterogeneous features.

To address these limitations, we propose GAC-Net, a Geometric–Attention Fusion Network that significantly improves depth completion performance by explicitly modeling 3D geometric priors and enhancing the multi-modal fusion mechanism. Specifically, GAC-Net incorporates two key modules. First, a dual-branch PointNet++-S encoder is designed with two parallel PointNet++ [28] branches of different receptive fields to extract scale-aware geometric features from the back-projected sparse point cloud. These features are fused using a channel attention mechanism [29] to form a robust global geometric representation. Second, a Channel Attention-Based Feature Fusion Module (CAFFM) is introduced to adaptively recalibrate and integrate the global 3D priors with RGB-depth features, improving the flexibility and accuracy of the fusion process.

The main contributions of this study are summarized as follows:

1. We propose a dual-branch PointNet++-S encoder to extract scale-aware geometric features from sparse point clouds and form a robust 3D representation;
2. We design a Channel Attention-Based Feature Fusion Module (CAFFM) to adaptively fuse geometric priors with RGB-depth features;
3. Extensive experiments on the KITTI depth completion benchmark demonstrate that GAC-Net outperforms BPNet and other published methods in both accuracy, robustness, and structural preservation, ranking first among peer-reviewed methods on the official leaderboard at the time of submission.

## 2. Related Work

### 2.1. Two-Dimensional-Based Depth Completion

Early deep learning methods for depth completion typically adopt a single-stage architecture [20,30,31], where sparse depth maps and RGB images are concatenated and processed by a shared convolutional encoder-decoder network. For example, the Sparse-to-Dense [31] framework proposed by Ma et al. fuses LiDAR inputs and monocular images through a unified backbone. Although these approaches are structurally simple and computationally efficient, they often produce overly smooth depth maps and fail to preserve fine geometric details around object boundaries.

To address these limitations, subsequent studies introduced two-stage frameworks [22,23,24,32,33,34,35,36,37], which first perform multi-modal feature fusion, followed by a refinement module for post-processing. CSPN [22] and CSPN++ [23], for instance, leverage convolutional spatial propagation networks to revisit the original sparse but accurate depth measurements and enhance edge sharpness. More recently, CFormer [32] extends this line of research by employing a CNN–Transformer hybrid encoder to extract and fuse RGB-depth features, followed by a refinement stage for fine-grained detail recovery. These methods gained popularity for their effectiveness in boundary preservation. However, directly applying convolution to sparse inputs compromises fusion quality, and this issue is also difficult to effectively resolve in the post-processing stage.

To further improve depth estimation quality, BPNet [26] proposes a three-stage architecture by adding a bilateral propagation module before fusion. This module generates an initial dense depth map from sparse input, providing structured geometric priors that alleviate data sparsity and enhance the overall completion performance, achieving state-of-the-art results at the time.

Nevertheless, all the above methods are fundamentally 2D-guided and lack explicit modeling of 3D spatial structure, which limits their ability to fully exploit the geometric information inherently embedded in sparse depth measurements.

### 2.2. 2D–3D Joint Depth Completion: Geometric, Multi-View, and Transformer Approaches

To better exploit the 3D structural information embedded in sparse depth data, recent studies [38,39,40,41,42,43,44,45,46,47,48] have explored three major directions: geometry-based approaches, multi-view/BEV representations, and Transformer-based cross-modal reasoning, each contributing complementary strengths to depth completion performance.

Geometry-based approaches can be further divided into three categories. The first category involves estimating surface normals as intermediate cues to ensure local geometric coherence. For instance, DeepLiDAR [38] and Depth-Normal [39] leverage normal estimation to improve local consistency during the completion process. The second category employs graph-based representations to capture geometric and contextual relationships. Typical works, such as ACMNet [40] and GraphCSPN [41], use graph neural networks to model spatial affinities and guide the effective fusion of RGB and depth features. The third category adopts point-based modeling by treating sparse LiDAR measurements as 3D point clouds. Notable examples include FuseNet [42] and PointDC [43], which directly extract 3D geometric features from the reprojected point cloud branch and combine them with image features for joint depth estimation.

Beyond explicit geometry, multi-view or bird’s-eye-view (BEV) representations are leveraged to capture global 3D structural consistency. For example, BEV@DC [44] constructs a BEV representation of sparse depth measurements to encode global spatial context while preserving fine-grained local geometry. More recently, TPVD [45] decomposes sparse depth maps into multiple perspective views that are subsequently reprojected and fused in 2D space, thereby reconstructing dense depth maps with improved global consistency.

Another emerging line of research explores Transformer-based architectures for cross-modal reasoning. The Dual-Attention Fusion model [46] employs a CNN–Transformer hybrid encoder and introduces a Dual-Attention Fusion module that combines convolutional spatial/channel attention with Transformer-based cross-modal attention. DeCoTR [47] further advances this line by formulating depth completion purely as a cross-modal Transformer reasoning problem, leveraging global attention to integrate RGB and depth features. More recently, HTMNet [48] extends this paradigm by coupling Transformer with a Mamba-based state-space bottleneck, representing the first attempt to introduce state-space modeling into depth completion and demonstrating promising results in handling challenging scenarios such as transparent and reflective objects.

Despite these advancements, existing methods still face several challenges. Geometry-based approaches often struggle to balance local precision and global consistency, multi-view/BEV-based representations increase computational cost, and Transformer-based frameworks, while powerful in modeling cross-modal relationships, generally involve complex architectures and heavy computational overhead. In contrast, our method adopts a point-based geometric encoding to explicitly exploit 3D structure and integrates it with an SE-based attention fusion module, thereby achieving a better balance between accuracy and efficiency for robust depth completion.

## 3. Methods

GAC-Net is designed to fully exploit the rich texture cues from RGB images and the accurate yet incomplete geometric priors from sparse depth data to generate high-quality dense depth maps. Let $I\in {\mathbb{R}}^{H\times W\times 3}$ be the input RGB image, $S\in {\mathbb{R}}^{H\times W\times 1}$ denotes the sparse depth map, and $D\in {\mathbb{R}}^{H\times W\times 1}$ denotes the predicted dense depth map.

**Figure 1:**
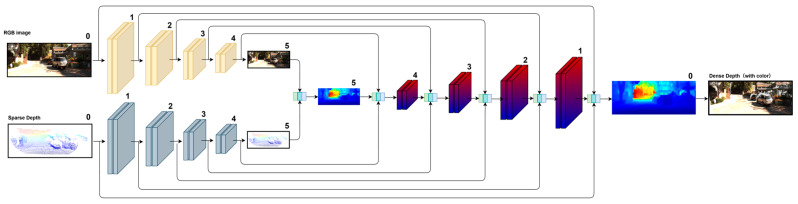
Overview of the multi-scale processing mechanism in GAC-Net.

**Figure 2:**
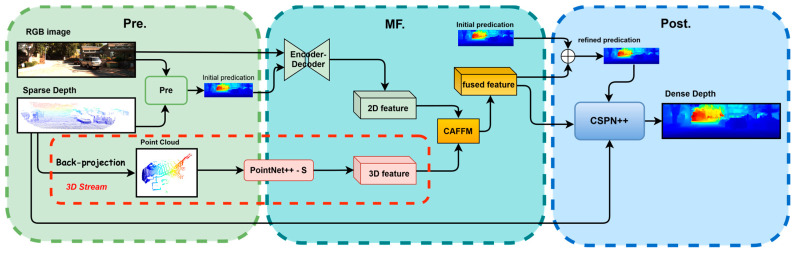
Overview of the three-stage network.

As illustrated in Figure 1 and Figure 2, GAC-Net builds upon the multi-scale, three-stage architecture proposed in BPNet [26], which performs coarse-to-fine depth prediction over six hierarchical scales (from scale-5 to scale-0). At each scale, depth prediction is performed in three sequential stages:

(1) Pre-processing stage: A bilateral propagation module generates an initial dense depth map from the sparse input, providing a more structured estimate for subsequent fusion;
(2) Enhanced multi-modal fusion: a U-Net backbone [27] extracts multi-scale 2D features, while the proposed dual-branch PointNet++-S encodes density-adaptive local and contextual 3D priors. These features are adaptively fused through the proposed Channel Attention-Based Feature Fusion Module (CAFFM), which reweights channels to enhance cross-modal consistency;
(3) Refinement stage: Finally, residual learning [49] and CSPN++ [23] are employed to iteratively refine the dense depth, enforcing local consistency and recovering fine-grained structures.

Figure 1 illustrates the progressive coarse-to-fine improvement of the depth map across scales, while Figure 2 shows the internal three-stage architecture within each resolution. The following sections present each module in detail (Section 3.1, Section 3.2 and Section 3.3) and describe the multi-scale training loss (Section 3.4).

### 3.1. Preprocessing via Bilateral Propagation

To generate a structured initial dense depth map from sparse input, we adopt the bilateral propagation module originally proposed by BPNet [26]. This module propagates valid depth measurements by dynamically aggregating neighboring pixels based on spatial proximity and radiometric similarity, effectively mitigating ambiguity caused by sparse sampling.

Given the input sparse depth map S, the initial dense depth $D^{0}$ can be computed as follows:

(1) $$D_{i}^{0}=\sum_{j\in N(i)}\omega_{ij}S_{j},\;\sum_{j}\omega_{ij}=1$$

Here, N(i) denotes the set of neighboring pixels for pixel i, and $\omega_{ij}$ are adaptive weights reflecting both spatial distance and color similarity.

This propagation provides a spatially structured initial estimate that serves as a reliable basis for the subsequent U-Net [27] encoding and the proposed PointNet++-S-based geometry extraction. It should be noted that, consistent with BPNet [26], the multi-scale coarse-to-fine strategy and the refinement stage remain unchanged, while our main contribution focuses on the 3D geometric modeling and enhanced multi-modal fusion modules described in Section 3.2 and Section 3.3.

### 3.2. Enhanced Multi-Modal Feature Fusion

**Figure 3:**
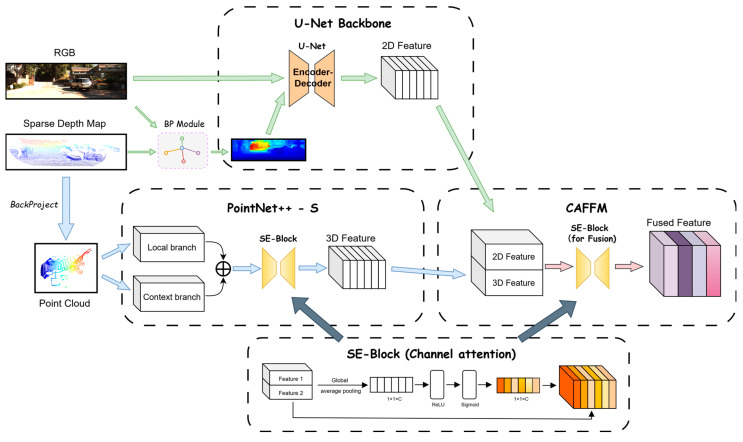
The architecture of the proposed 2D–3D feature extraction and fusion pipeline. The pipeline consists of a U-Net backbone for multi-scale 2D feature extraction, a dual-branch PointNet++-S module for density-adaptive 3D geometric modeling from back-projected point clouds, and a Channel Attention-Based Feature Fusion Module (CAFFM) for adaptive integration of 2D and 3D features. SE-Blocks are employed in both 3D encoding and feature fusion stages to enhance channel-wise interactions.

To further enhance the robustness and accuracy of depth completion, GAC-Net improves the conventional multi-modal fusion design by explicitly incorporating global 3D geometric priors and a channel attention mechanism for adaptive cross-modal integration. Specifically, the proposed module combines a U-Net [27] backbone for multi-scale 2D feature extraction, a dual-branch PointNet++-S encoder for multi-scale 3D geometry representation, and a Channel Attention-Based Feature Fusion Module (CAFFM) to adaptively fuse the 2D and 3D features. The detailed structure of these components and their interaction is shown in Figure 3.

#### 3.2.1. U-Net Backbone for 2D Feature Fusion

In the multi-modal fusion stage, the RGB image I and the initial dense depth map $D^{0}$ generated by the bilateral propagation module are concatenated to form the 2D input tensor:

(2) $$X_{\mathrm{in}}=\mathrm{Concat}\left(I,D^{0}\right)$$

A U-Net [27] encoder-decoder backbone (encoder-decoder network) is then employed to extract hierarchical multi-scale 2D features $F_{2D}$:

(3) $$F_{2D}=\mathrm{U-Net}\left(X_{\mathrm{in}}\right)$$

This architecture progressively captures local texture, edge, and structural context through down-sampling and restores spatial resolution with skip connections during up-sampling. The resulting feature maps provide rich 2D cues for robust multi-modal fusion.

#### 3.2.2. Dual-Branch PointNet++-S Encoder for 3D Geometry Representation

To capture expressive geometric features and maintain robustness under varying sparsity conditions, we propose a dual-branch PointNet++-S encoder, where S stands for Scale-aware, indicating its ability to extract multi-scale 3D representations from sparse depth inputs.

Let $P=\{p_{i}\in {\mathbb{R}}^{3}|i=1,\ldots ,N\}$ denote the 3D point cloud obtained by back-projecting valid sparse depth pixels using camera intrinsics. The encoder consists of two parallel PointNet++ [28] branches with distinct receptive field sizes, designed to extract both local and contextual geometric features.

The local branch aggregates fine-grained neighborhood information via two Set Abstraction (SA) layers:

(4) $$F_{\mathrm{local}}^{\left(1\right)}=SA_{L_{1}}\left(P;384,0.2m,32\right)$$

(5) $$F_{\mathrm{local}}^{\left(2\right)}=SA_{L_{2}}\left(F_{\mathrm{local}}^{\left(1\right)};128,0.4m,64\right)$$

where each SA layer samples a fixed number of neighboring points within a predefined radius.

Specifically, each Set Abstraction (SA) layer is defined by a tuple (n, r, k), where the following is true:

- n is the number of center points sampled from the point cloud using farthest point sampling (FPS);
- r is the ball query radius used to group neighboring points around each center;
- k is the number of neighboring points selected within radius r for local feature aggregation.

These parameters control the receptive field and granularity of geometric information captured at each SA layer.

The context branch uses larger receptive fields to model broader scene-level geometry:

(6) $$F_{\mathrm{context}}^{\left(1\right)}=SA_{C_{1}}\left(P;256,0.4m,32\right)$$

(7) $$F_{\mathrm{context}}^{\left(2\right)}=SA_{C_{2}}\left(F_{\mathrm{context}}^{\left(1\right)};64,0.6m,64\right)$$

The outputs from both branches are concatenated to form a unified multi-scale 3D feature:

(8) $$F_{3D}=\mathrm{Concat}\left(F_{\mathrm{local}}^{\left(2\right)},F_{\mathrm{context}}^{\left(2\right)}\right)\in {\mathbb{R}}^{256}$$

To emphasize informative channels, we apply a squeeze-and-excitation (SE) block [29] for channel-wise recalibration. The SE block first performs global average pooling:

(9) $$z_{c}=\frac{1}{N}\sum_{i=1}^{N}F_{3D}^{\left(c\right)}(i),\;c=1,\ldots ,256$$

This channel descriptor is passed through two fully connected layers with ReLU and Sigmoid activations to compute attention weights:

(10) $$s=\sigma \left(W_{2}\delta (W_{1}z)\right)$$

The recalibrated global 3D feature is then obtained via channel-wise scaling:

(11) $$G_{3D}=s⊙F_{3D}$$

where ⊙ denotes element-wise multiplication. Finally, $G_{3D}$ is broadcasted spatially to match the dimensions of the 2D feature map for cross-modal fusion in the next stage.

#### 3.2.3. Channel Attention-Based Feature Fusion Module (CAFFM)

To integrate complementary local 2D texture features and global 3D geometric priors, we propose a Channel Attention-Based Feature Fusion Module (CAFFM). Let $F_{2D}\in {\mathbb{R}}^{H\times W\times c_{2}}$ denote the multi-scale 2D feature map extracted by the U-Net [27] backbone, and $G_{3D}\in {\mathbb{R}}^{c_{3}}$ represent the recalibrated global 3D feature vector obtained from the PointNet++-S encoder.

To enable effective fusion, the 3D feature is first broadcast spatially and concatenated with the 2D feature along the channel dimension:

(12) $$F_{\mathrm{fusion}-\mathrm{in}}=\mathrm{Concat}\left(F_{2D},\;{\tilde{G}}_{3D}\right),\;F_{\mathrm{fusion}-\mathrm{in}}\in {\mathbb{R}}^{H\times W\times (c_{2}+c_{3})}$$

where ${\tilde{G}}_{3D}$ denotes the spatially broadcasted version of $G_{3D}$.

A squeeze-and-excitation (SE) block [29] is then applied to adaptively learn the fusion attention. First, global average pooling compresses spatial information across all channels:

(13) $$z_{c}=\frac{1}{HW}\sum_{i=1}^{H}\sum_{j=1}^{W}F_{\mathrm{fusion}-\mathrm{in}}^{\left(c\right)}(i,j),\;c=1,\ldots ,C$$

where $C\;=\;c_{2}+c_{3}$.

This channel descriptor is passed through two fully connected layers with ReLU and Sigmoid activations to produce the channel attention weights:

(14) $$s=\sigma \left(W_{2}\delta (W_{1}z)\right)$$

The final fused feature map is recalibrated via channel-wise scaling:

(15) $$F_{\mathrm{fusion}}=s⊙F_{\mathrm{fusion}-\mathrm{in}}$$

where ⊙ denotes element-wise multiplication across channels.

This adaptively fused representation is subsequently passed to the refinement stage to generate the final high-quality dense depth map.

#### 3.2.4. Pseudocode for Enhanced Multi-Modal Feature Fusion

To provide a concise overview of the proposed fusion mechanism, we summarize the workflow in Algorithm 1. At a given scale s, the inputs include the RGB image I, the sparse depth D, and the pre-processed dense depth $D_{s}^{\left(\mathrm{pre}\right)}$ obtained from the bilateral propagation (Section 3.1). The process first extracts 3D features from the back-projected point cloud using a dual-branch PointNet++-S with SE-based recalibration, while 2D features are encoded from the concatenated RGB-depth input using a U-Net encoder. Next, the 3D features are spatially broadcast to the image grid and fused with the 2D features through the CAFFM module with channel-attention weighting, producing the enhanced multi-modal feature $F_{s}$. This procedure corresponds to Equations (2)–(15), and its pseudocode implementation is provided in Algorithm 1.
**Algorithm 1:** Enhanced Multi-Modal Feature Fusion at scale 
s (Equations (2)–(15))
**Input:** RGB image $I\in R^{H\times W\times 3}$
 Sparse depth map $D\in R^{H\times W\times 1}$
 Pre-processed dense depth (from BP) $D_{s}^{\left(pre\right)}\in R^{H\times W}$ Camera intrinsics $K\in R^{3\times 3}$ **Output:** Fused feature map $F_{s}\in R^{H\times W\times C}$
**Notion:**
 ⊕: channel concatenation;

 ⊙: channel-wise multiplication;
 GAP: global average pooling;
 δ(⋅): ReLU;
 σ(⋅): Sigmoid
 $D_{s}^{\left(pre\right)}$: dense depth pre−processed via Bilateral Propagation **Procedure:** 1: Step 1: Back-projection to 3D 2:  Construct sparse point cloud $P=BackProject\left(S,K\right)$. 3: Step 2: 2D feature encoding 4:  Form 2D input $X=[I⊕D_{s}^{\left(pre\right)}]$ 5:  $F_{2D}=U-Net\left(X\right)$ 6: Step 3: Dual-branch PointNet++-S 7:  For each branch $b\in \left\{local,context\right\}$**:**
8:   For each SA layer with config $\left(n_{l},r_{l},k_{l}\right)$:
9:    $g_{l}^{\left(b\right)}\leftarrow \mathrm{PointNetSA}(P;n_{l},r_{l},k_{l})$
10:   End for 11:  End for
12:  Aggregate to multi-scale 3D feature $F_{3D}=\mathrm{Concat}\left(F_{\mathrm{local}}^{\left(2\right)},F_{\mathrm{context}}^{\left(2\right)}\right)$ 13: Step 4: Channel recalibration on 3D 14:  $z=\mathrm{GAP}\left(F_{3D}\right)$; 15:  $s=\sigma \left(W_{2}\cdot \delta \left(W_{1}z\right)\right)$; 16:  $G_{3D}=s⊙F_{3D}$. 17: Step 5: Spatial broadcasting of 3D feature 18:  $\tilde{G_{3D}}=\mathrm{Broadcast}\left(G_{3D}\to H\times W\right)$
19: Step 6: CAFFM: channel-attention fusion 20:  $F_{\mathrm{in}}=\mathrm{Concat}\left(F_{2D},\tilde{G_{3D}}\right)$; 21:  $z=\mathrm{GAP}\left(F_{\mathrm{in}}\right)$; 22:  $\alpha =\sigma \left(W_{2}\cdot \delta \left(W_{1}z\right)\right)$); 23:  $F_{s}=\alpha ⊙F_{\mathrm{in}}$. 24: **return** $F_{s}$.

### 3.3. Depth Refinement

Although the fused depth map captures most scene structures, it may still suffer from local inconsistencies and blurred boundaries. To address this, we adopt a combination of residual learning [49] and CSPN++ [23] in the refinement stage.

Specifically, residual learning [49] corrects global prediction errors using the fused feature $F_{\mathrm{fused}}$:

(16) $$D_{\mathrm{res}}=D^{*}+R(F_{\mathrm{fused}})$$

where $R\left(\;\cdot \;\right)$ denotes the residual regressor. Subsequently, CSPN++ [23] refines local details by iteratively propagating depth values:

(17) $$D_{i}^{t+1}=\sum_{j\in N(i)}\kappa_{ij}D_{j}^{t}+\left(1-\sum_{j}\kappa_{ij}\right)S_{i}$$

where $\kappa_{ij}$ are learned affinity weights.

This refinement process preserves clear depth boundaries, improves the structural integrity of ambiguous regions, and enhances the overall accuracy of the predicted depth map, thereby supporting more precise estimation in the subsequent stages of the multi-scale framework.

### 3.4. Loss Function

To supervise the training of GAC-Net in an end-to-end manner, we adopt a multi-scale hybrid loss that combines the mean squared error (L2) and the mean absolute error (L1) terms, providing robust guidance for both global consistency and local structural accuracy. Specifically, for each predicted depth map at scale s, we apply a bilinear up-sampling operator to match the ground truth resolution and compute the loss within valid pixel regions. The total training loss is defined as follows:

(18) $$L=\sum_{s=0}^{5}\lambda_{s}\sum_{i\in P}\left({\left|D_{i}^{g}-U_{s}(D^{*})\right|}_{2}^{2}+{\left|D_{i}^{g}-U_{s}(D^{s})\right|}_{1}\right)$$

where $D^{*}$ denotes the ground truth dense depth map, $D^{s}$ is the predicted depth at scale s, $U_{s}(\cdot )$ is the bilinear up-sampling operator, P is the set of valid pixels in $D^{g}$, and $\lambda_{s}$ is a scale-dependent weight set to $4^{-s}$, following the setting in BPNet [26].

This hybrid loss ensures that GAC-Net effectively minimizes both global depth estimation errors and local fine-grained residuals across multiple resolutions, producing high-quality dense depth maps even under sparse and noisy input conditions.

## 4. Experiment

### 4.1. Experiment Setting

#### 4.1.1. Datasets

All experiments are conducted on the KITTI Depth Completion (DC) benchmark [18], which is widely used to evaluate outdoor depth completion performance. The dataset is collected from an autonomous driving platform, with the ground truth depth generated by temporally registering multiple LiDAR scans and refined using stereo image pairs. It consists of 86,898 training frames, 1000 validation frames, and 1000 test frames. Following common practice, we crop the upper 100-pixel region (where LiDAR data is typically missing) from the original resolution of 1216 × 352 to 1216 × 256 during training. In inference, the full resolution is used for evaluation.

#### 4.1.2. Training Details

We implement GAC-Net using PyTorch and train it on a workstation equipped with four NVIDIA RTX 4090 GPUs. The sparse depth map is first back-projected and processed with the proposed dual-branch PointNet++-S encoder to extract a robust global geometric feature.

Following common practice in recent depth completion works [26,50], we use the AdamW optimizer with a weight decay of 0.05 and apply gradient clipping with a maximum L2 norm of 0.1. The learning rate is scheduled using a warm-up and cosine annealing strategy: it starts at 1/40 of the maximum value (2.5 × 10^–4^), linearly increases to the maximum in the first 10% of iterations, and then decays to 10% of the maximum following a cosine curve for the remaining 90% of training. The batch size is set to 2 per GPU. The model is trained for 30 epochs. Random brightness and contrast augmentation is applied to RGB images during training.

**Figure 4:**
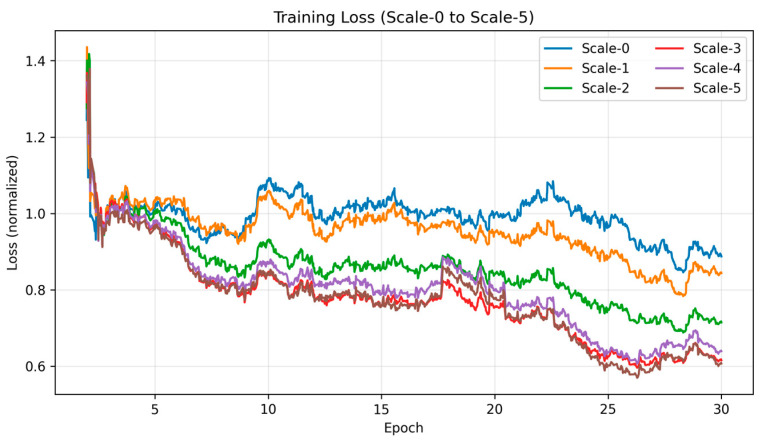
Normalized training curves of the per-scale losses (Scale-0 to Scale-5) during training. All curves decrease consistently and converge to a stable plateau after sufficient iterations.

**Figure 5:**
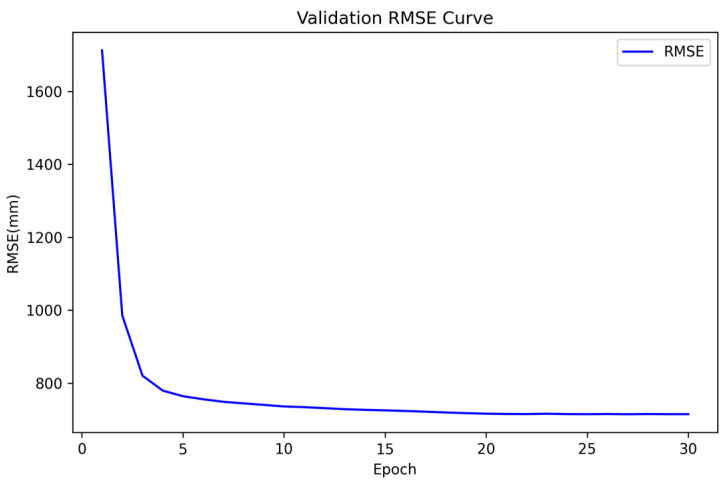
Validation curve of the main evaluation metric, RMSE. The curve decreases smoothly and reaches a stable plateau after approximately 20 epochs.

To further illustrate the convergence process, Figure 4 presents the normalized training curves of the per-scale losses (Scale-0 to Scale-5), which are plotted together for clarity. All curves consistently decrease and converge to a stable plateau after sufficient iterations. Figure 5 shows the validation curve of the main evaluation metric (RMSE), which decreases smoothly and stabilizes after approximately 20 epochs.

#### 4.1.3. Metrics

Four standard metrics are used to evaluate performance: root mean squared error (RMSE, in mm), mean absolute error (MAE, in mm), root mean squared error of inverse depth (iRMSE, in 1/km), and mean absolute error of inverse depth (iMAE, in 1/km). Among these, RMSE is the primary metric used for ranking on the official KITTI benchmark.

The following formula shows the detailed definition of each indicator:

(19) $$\mathrm{RMSE}=\sqrt{\frac{1}{m}\sum_{i=1}^{m}{\left(y_{i}-y_{gt}\right)}^{2}}$$

(20) $$\mathrm{MAE}=\frac{1}{m}\sum_{i=1}^{m}\left|y_{i}-y_{gt}\right|$$

(21) $$\mathrm{iRMSE}=\sqrt{\frac{1}{m}\sum_{i=1}^{m}{\left(\frac{1}{y_{i}}-\frac{1}{y_{gt}}\right)}^{2}}$$

(22) $$\mathrm{iMAE}=\frac{1}{m}\sum_{i=1}^{m}\left|\frac{1}{y_{i}}-\frac{1}{y_{gt}}\right|$$

### 4.2. Comparison with SoTA Methods

To comprehensively validate the superiority of the proposed GAC-Net, we conduct comparison experiments from both quantitative and qualitative perspectives.

#### 4.2.1. Quantitative Comparison

Table 1 summarizes the performance of GAC-Net and representative state-of-the-art (SOTA) methods. The upper section lists purely 2D-based approaches, while the lower section includes 2D–3D joint methods that incorporate geometric priors from LiDAR point clouds.

Our GAC-Net achieves the lowest RMSE of 680.82 mm, surpassing BPNet [26] (684.90 mm), TPVD [45] (693.97 mm), and other recent methods. This result demonstrates the effectiveness of our enhanced multi-modal fusion framework, particularly the integration of scale-aware 3D geometry via PointNet++-S and adaptive channel-wise fusion through CAFFM.

In addition to RMSE, GAC-Net also achieves competitive performance on other metrics, including MAE, iRMSE, and iMAE, indicating both global accuracy and robustness in local structures. Notably, GAC-Net ranks first among all peer-reviewed methods on the official KITTI leaderboard at the time of submission.

In contrast to early 2D-based methods such as CSPN++ [23] and PENet [21], which primarily rely on image-guided convolutions, GAC-Net leverages explicit 3D geometric encoding to address the irregularity and sparsity of LiDAR inputs. While 2D methods can effectively propagate depth in high-texture regions, they often fail to capture reliable geometric structures in textureless or occluded areas, leading to blurred or distorted reconstructions. By integrating point-based representations through the PointNet++-S encoder, our model significantly improves structural completeness and robustness in such challenging scenarios.

Compared with recent 2D–3D joint methods such as TPVD [45] and DeCoTR [47], GAC-Net adopts a more modular and geometry-driven design. TPVD [45] decomposes the sparse input into multiple perspective views to enhance structural awareness, but it also introduces repeated projection steps, which tend to increase error accumulation and processing complexity. DeCoTR [47], on the other hand, integrates 2D and 3D features via global attention, facilitating long-range interactions across modalities. However, both approaches involve complex processing pipelines. In contrast, GAC-Net focuses on scale-aware point-based geometry encoding and adaptive channel-wise fusion, yielding lower RMSE while maintaining architectural clarity and generalizability across varying input sparsity levels.

#### 4.2.2. Qualitative Comparison

**Figure 6:**
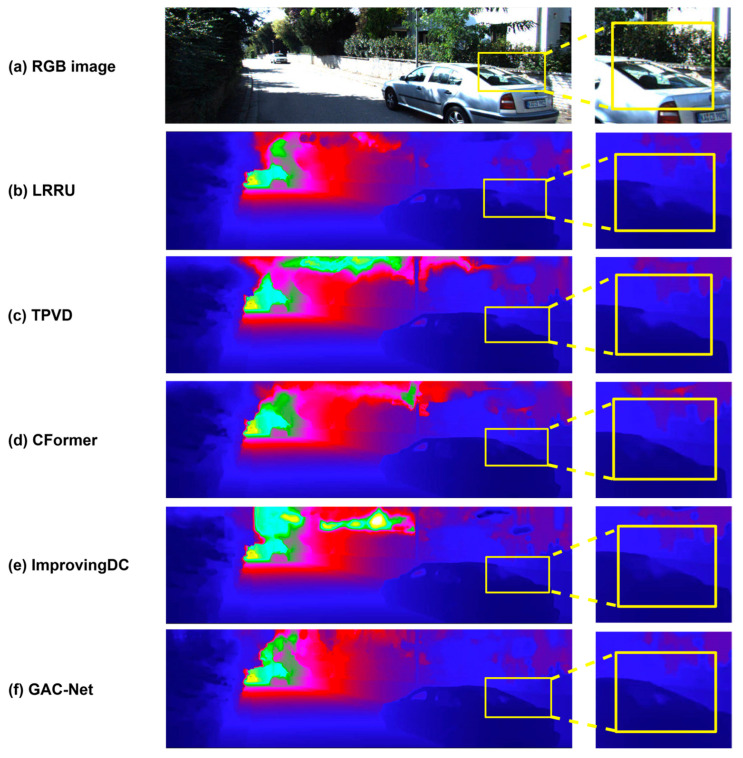
Qualitative comparison with state-of-the-art methods (LRRU [52], TPVD [45], CFormer [32] and ImprovingDC [50]) on preserving the structural completeness of objects, highlighting the reconstruction of full vehicles (e.g., rear windshield and boundary contours) under sparse input.

**Figure 7:**
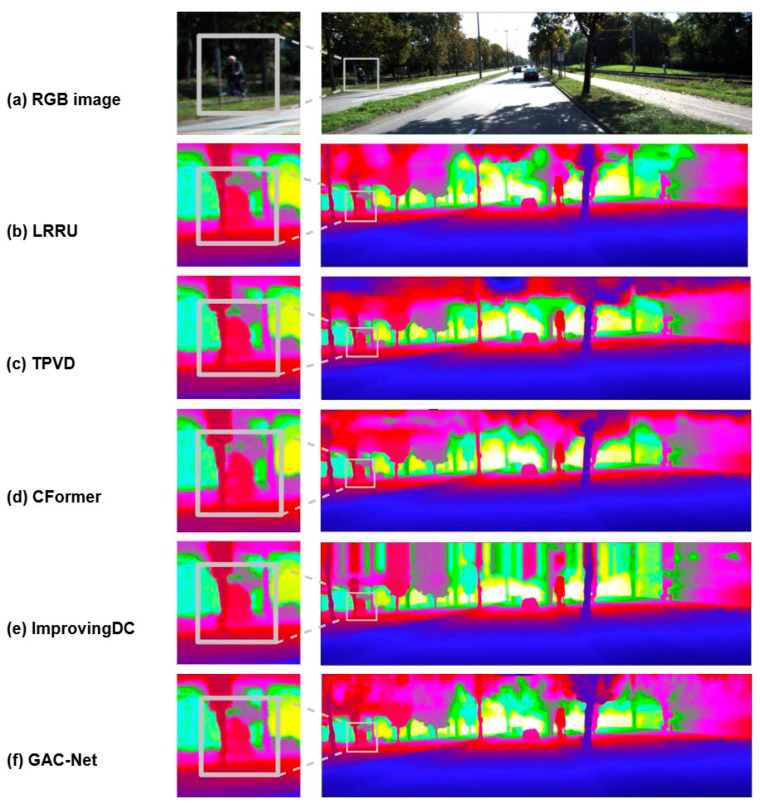
Visual comparison of local structural details among state-of-the-art methods (LRRU [52], TPVD [45], CFormer [32] and ImprovingDC [50]), with a focus on edge sharpness and boundary preservation for objects such as traffic signs and cyclists.

**Figure 8:**
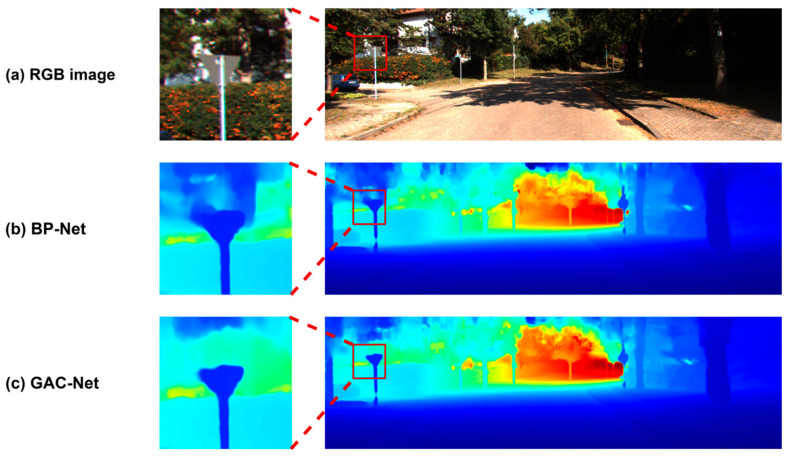
Visual comparison between BPNet [26] and our GAC-Net.

We further conduct visual comparisons to assess the perceptual quality of the predicted depth maps. As shown in Figure 6, Figure 7 and Figure 8, GAC-Net is compared with four representative state-of-the-art methods: LRRU [52], TPVD [45], CFormer [32], and ImprovingDC [50], as well as its baseline model BPNet [26].

Figure 6 focuses on the reconstruction of large-scale foreground objects, particularly a complete vehicle. The zoom-in region highlights GAC-Net’s ability to recover the rear windshield and its boundary with higher completeness and clarity, while other methods either produce incomplete structures (e.g., LRRU [52]) or blurred and collapsed outlines (e.g., TPVD [45] and CFormer [32]). This illustrates GAC-Net’s superior capability in handling spatially sparse but structurally significant areas.

Figure 7 compares the preservation of fine structures such as thin poles and distant pedestrians. In the zoomed views, GAC-Net demonstrates sharper edges and better geometric integrity, whereas other methods show common issues such as edge blurring, structural breakage, or background fusion. For instance, TPVD [45] exhibits strong edge diffusion, and ImprovingDC [50] fails to recover the basic shape of the pedestrian’s head. These results indicate that GAC-Net can effectively retain high-frequency details even under highly sparse input conditions.

Figure 8 provides a direct comparison between GAC-Net and BPNet [26], verifying the practical improvements brought by our proposed modules. In the zoom-in regions, GAC-Net exhibits sharper, more continuous, and more complete reconstructions of edge structures such as street signs and tree trunks. By contrast, BPNet [26] suffers from elongated contours and edge spreading. These differences confirm that the incorporation of 3D geometric encoding and channel-aware fusion in GAC-Net significantly enhances its ability to reconstruct complex scene elements.

In summary, the differences observed in the zoom-in regions clearly demonstrate that GAC-Net achieves more complete, sharper, and geometrically consistent depth estimation results across various object types, including large foreground objects (e.g., vehicles), mid-scale structures (e.g., poles), and fine, distant elements (e.g., pedestrians).

### 4.3. Ablation Studies

To comprehensively evaluate the contributions of each core module in GAC-Net, we conduct ablation studies on the KITTI validation set [18]. Specifically, we analyze the impact of the geometric encoding module (PointNet++-S) and the channel-aware feature fusion module (CAFFM). The experimental results are summarized in Table 2 and Table 3, respectively.

#### 4.3.1. Effectiveness of PointNet++-S

Table 2 shows the performance gain brought by incorporating the PointNet++-S module. Starting from a baseline model (i) that only adopts the original multi-modal fusion pipeline without any 3D encoder or attention mechanism, we observe an RMSE of 719.08 mm and an MAE of 204.36 mm. By adding a standard PointNet++ encoder (ii), the performance improves to 714.34 mm RMSE and 200.12 mm MAE, confirming the benefit of introducing explicit 3D geometric priors. Furthermore, replacing the vanilla encoder with our proposed dual-branch PointNet++-S module (iii) leads to the best performance (RMSE: 711.40 mm, MAE: 197.63 mm), demonstrating the effectiveness of the scale-aware geometric extraction strategy.

#### 4.3.2. Effectiveness of CAFFM

As shown in Table 3, we investigate different feature fusion strategies to validate the advantage of our CAFFM module. The baseline model (i) uses element-wise addition for combining local and contextual features, yielding 716.69 mm RMSE and 202.54 mm MAE. Switching to channel-wise concatenation (ii) slightly improves the performance to 715.06 mm RMSE and 201.36 mm MAE. Finally, applying our proposed channel-aware feature fusion module (CAFFM) (iii) further reduces the RMSE to 711.40 mm and MAE to 197.63 mm. This confirms that CAFFM enables richer feature interactions and enhances overall depth completion accuracy.

#### 4.3.3. Visual Comparison of Ablation Results

**Figure 9:**
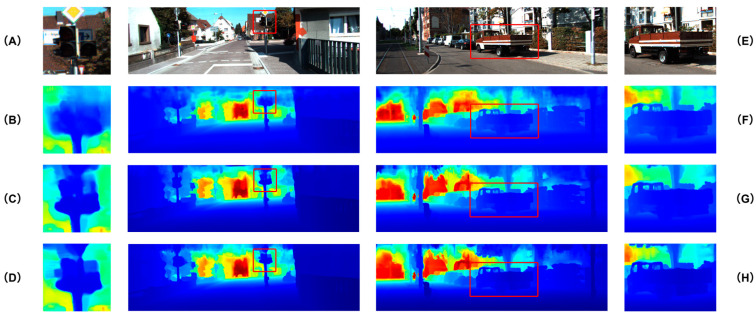
Visual comparison of ablation experiments on the KITTI validation set [18]. (**A**–**D**) show a local region around a traffic sign and its background, while (**E**–**H**) present a large-scale scene containing a vehicle. From top to bottom: RGB input image (**A**,**E**), baseline model without PointNet++-S and CAFFM (**B**,**F**), variant with PointNet++-S but simple concatenation instead of CAFFM (**C**,**G**), and the full GAC-Net with both PointNet++-S and CAFFM (**D**,**H**).

To complement the quantitative results in Table 2 and Table 3, Figure 9 provides visual comparisons of ablation experiments on representative KITTI samples. As shown on the left (A–D), the baseline model without PointNet++-S and CAFFM produces blurry boundaries, where the traffic sign is merged with the background. Adding PointNet++-S improves geometric consistency, but without CAFFM, the edges remain less distinct. The complete GAC-Net successfully separates the sign from the background, yielding sharper and clearer boundaries. On the right (E–H), a larger-scale scene further demonstrates that both PointNet++-S and CAFFM contribute to reconstructing the complete structure of the vehicle, with the full GAC-Net achieving the most accurate and visually coherent depth maps. These qualitative results are consistent with the quantitative improvements reported in the ablation study.

### 4.4. Sparsity Level Analysis

To assess the robustness of our method under varying input sparsity levels, we simulate different degrees of depth sparsity by uniformly sampling valid pixels from the original sparse depth maps. Following standard practice [26,45], the sparsity ratio is varied from 0.4 to 1.0, where 1.0 corresponds to the original input sparsity.

**Figure 10:**
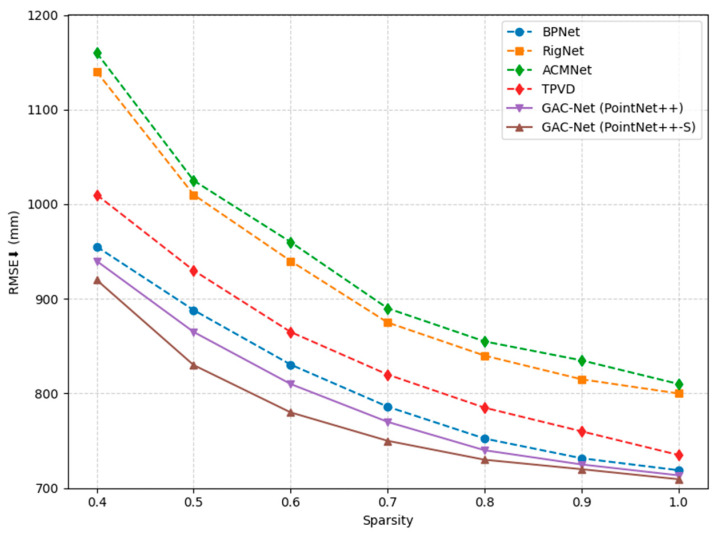
Comparison of RMSE (mm) across different input depth sparsity levels.

The results are presented in Figure 10, where our GAC-Net variants are compared against three representative methods: BPNet [26], TPVD [45], RigNet [35], and ACMNet [40]. As expected, all methods suffer a noticeable performance drop in RMSE as the input becomes sparser. However, both versions of GAC-Net consistently outperform other approaches across all sparsity levels.

Notably, GAC-Net (PointNet++–S) achieves the lowest RMSE in every case, especially under more challenging sparsity conditions (e.g., 0.4–0.6). This performance validates the effectiveness of our scale-aware dual-branch PointNet++–S module, which captures both local geometric detail and global spatial structure. These results confirm that our method maintains higher accuracy and stronger generalization ability in sparse depth completion tasks under real-world constraints.

### 4.5. Complexity Analysis

To further evaluate the efficiency of our framework, we compare the model complexity of GAC-Net with BPNet [26] and the Transformer-based Dual-Attention Fusion model [46]. The results are summarized in Table 4. As shown, our method contains 91.07 M parameters, which is slightly higher than BPNet [26] (89.87 M) due to the additional PointNet++-S branch for 3D geometric feature extraction. This design, however, only introduces a marginal increase in inference time (0.17 s vs. 0.16 s), demonstrating that the added geometric modeling branch does not significantly impact efficiency.

Compared with the Transformer-based Dual-Attention Fusion model, GAC-Net runs faster (0.17 s vs. 0.191 s), despite having more parameters (91.07 M vs. 31.38 M). This advantage mainly stems from the lightweight SE-based CAFFM fusion module, which effectively avoids the runtime overhead introduced by Transformer modules.

Overall, the results indicate that GAC-Net achieves a favorable balance between accuracy and efficiency. Although not strictly real-time, the design demonstrates that incorporating 3D modeling through the PointNet++-S branch and the SE-based CAFFM fusion module can be achieved with only marginal computational overhead.

## 5. Conclusions

This paper presented GAC-Net, a geometric attention fusion framework for image-guided depth completion. Building on the multi-scale, three-stage pipeline of BPNet, GAC-Net introduces two key modules: (i) a dual-branch PointNet++-S encoder that extracts scale-aware 3D geometric features from back-projected sparse point clouds and (ii) a Channel-Attention-Based Feature Fusion Module (CAFFM) that adaptively integrates geometric priors with RGB-depth features. These designs enhance explicit 3D modeling and cross-modal alignment, leading to improved preservation of both global structures and fine details. On the KITTI benchmark, GAC-Net demonstrates competitive performance among peer-reviewed methods, achieving accuracy improvements in both global structures and local details.

We further analyzed model complexity and showed that, while GAC-Net includes an additional 3D branch, the overall inference overhead remains marginal and indicates a favorable trade-off between accuracy and efficiency compared with Transformer-based baselines.

Limitations and future work:

(1) Efficiency under deployment constraints. Although not strictly real-time, our results indicate that incorporating 3D modeling through the PointNet++-S branch and the SE-based CAFFM fusion module can be achieved with only modest computational overhead. Future work will explore hardware-friendly acceleration (e.g., TensorRT, mixed precision), model compression (pruning/quantization/distillation), and lightweight design choices (e.g., streamlined multi-scale stages and operator fusion) to further reduce latency and memory footprint. We will also investigate efficient cross-modal reasoning modules (e.g., state-space models such as Mamba or linear-attention variants) under strict efficiency constraints.
(2) Generalization beyond KITTI. To strengthen external validity, we plan to evaluate cross-dataset performance and domain transfer (e.g., additional outdoor/indoor benchmarks) and study robustness to sparsity, noise, transparency/reflectivity, and calibration shifts. In particular, recent benchmarks such as RSUD20K highlight the importance of dataset diversity and robustness evaluation under diverse environmental conditions, which will inspire our future exploration of generalization across challenging scenarios.
(3) Temporal and system aspects. We will extend GAC-Net to sequential/streaming depth completion with temporal consistency and conduct system-level measurements (FLOPs and inference memory on diverse hardware, including embedded platforms), providing a more comprehensive view of computational costs for practical deployment.

## Figures and Tables

**Table 1:** Quantitative result on KITTI dataset.

Method	2D	3D	RMSE↓	MAE↓	iRMSE↓	iMAE↓	Publication
CPSN [22]	✓		1019.64	279.46	2.93	1.15	ECCV 2018
TWISE [51]	✓		840.20	195.58	2.08	0.82	CVPR 2021
CSPN++ [23]	✓		743.69	209.28	2.07	0.90	AAAI 2020
PENet [21]	✓		730.08	210.5	2.17	0.94	ICRA 2021
RigNet [35]	✓		712.66	203.25	2.08	0.90	ECCV 2022
LRRU [52]	✓		696.51	189.96	1.87	0.81	ICCV 2023
BP-Net [26]	✓		684.90	194.69	1.82	0.84	CVPR 2024
FuseNet [42]	✓	✓	752.88	221.19	2.34	1.14	ICCV 2019
ACMNet [40]	✓	✓	744.91	206.09	2.08	0.90	T-IP 2021
PointFusion [53]	✓	✓	741.90	201.10	1.97	0.85	ICCV 2021
GraphCSPN [41]	✓	✓	738.41	199.31	1.96	0.84	ECCV 2022
PointDC [43]	✓	✓	736.07	201.87	1.97	0.87	ICCV 2023
DeCoTR [47]	✓	✓	717.07	195.30	1.92	0.84	CVPR 2024
TPVD [45]	✓	✓	693.97	**188.60**	1.82	**0.81**	CVPR 2024
GAC-Net(ours)	✓	✓	**680.82**	193.85	**1.81**	0.84	-

↓ indicates that a lower value is better. The optimal numerical results are highlighted in bold.

**Table 2:** Ablation studies on PointNet++-S.

GAC-Net	Without	PointNet++	PointNet++-S	RMSE	MAE
i	✓			719.08	204.36
ii		✓		714.34	200.12
iii			✓	709.4	195.63

**Table 3:** Ablation studies on CAFFM.

GAC-Net	Add	Concat	CAFFM	RMSE	MAE
i	✓			716.69	202.54
ii		✓		715.06	201.36
iii			✓	709.4	195.63

**Table 4:** Comparison of model complexity.

Method	Publication	Params (M)	Inference Time (s)
BPNet [26]	CVPR 2024	89.87	0.160
Dual-Attention Fusion [46]	Sensors 2024	31.38	0.191
Ours	-	91.07	0.170

## Data Availability

Data is contained within the article.
